# Exergy and Exergoeconomic Analysis of a Cogeneration Hybrid Solar Organic Rankine Cycle with Ejector

**DOI:** 10.3390/e22060702

**Published:** 2020-06-24

**Authors:** Bourhan Tashtoush, Tatiana Morosuk, Jigar Chudasama

**Affiliations:** 1 Mechanical Engineering Department, Jordan University of Science and Technology, Irbid 22110, Jordan; bourhan@just.edu.jo; 2 Institute for Energy Engineering, Technische Universität Berlin, Marchstr. 18, 10587 Berlin, Germany; j.chudasama@campus.tu-berlin.de

**Keywords:** exergy analysis, economic analysis, exergoeconomic analysis, ejector refrigeration cycle, organic Rankine cycle

## Abstract

Solar energy is utilized in a combined ejector refrigeration system with an organic Rankine cycle (ORC) to produce a cooling effect and generate electrical power. This study aims at increasing the utilized share of the collected solar thermal energy by inserting an ORC into the system. As the ejector refrigeration cycle reaches its maximum coefficient of performance (COP), the ORC starts working and generating electrical power. This electricity is used to run the circulating pumps and the control system, which makes the system autonomous. For the ejector refrigeration system, R134a refrigerant is selected as the working fluid for its performance characteristics and environmentally friendly nature. The COP of 0.53 was obtained for the ejector refrigeration cycle. The combined cycle of the solar ejector refrigeration and ORC is modeled in EBSILON Professional. Different parameters like generator temperature and pressure, condenser temperature and pressure, and entrainment ratio are studied, and the effect of these parameters on the cycle COP is investigated. Exergy, economic, and exergoeconomic analyses of the hybrid system are carried out to identify the thermodynamic and cost inefficiencies present in various components of the system.

## 1. Introduction

The conventional vapor-compression refrigeration system (VCRS), which uses working fluids that are harmful to the environment, is dominating in the refrigeration sector all over the world. The solar ejector refrigeration system (SERS), which uses solar energy as the driving energy, is the alternative for VCRS. Thus, it helps to decrease the indirect environmental impact though reducing the CO_2_ emissions coming from electricity generation. The maintenance cost required of the ejector refrigeration system (ERS) is meager [1]. Solar energy is the most abundant, vast, and inexhaustible source of energy for a clean environment. Researchers have started investigating the replacement of high-priced fossil fuels with alternative renewable energy sources such as wind, solar, and geothermal. As the supply of solar energy is highly compatible with the demand, the use and implementation of solar energy in air conditioning and refrigeration applications have gained more attention over the last decades.

The conventional solar cooling and refrigeration cycles include sorption machines such as adsorption and absorption machines, or desiccant wheels. Furthermore, SERS is competitive with the mentioned technologies as they have lower cost, no moving parts, simple system design, and can be driven by low-grade thermal sources [1,2]. The solar energy can be used to drive an organic Rankine cycle (ORC) to generate electrical power [3,4,5]. Combined cycles and systems were proposed by several researchers to maximize the utilization of wasted heat and renewable energy and reduce fossil fuel consumption to alleviate environmental problems [6].

Many researchers have investigated the combined ERS with ORC for the production of cold and power [7,8,9]. Li et al. [10] proposed an ORC with an ejector (EORC) to increase the power output capacity and cycle efficiency. Exergy and energy analyses of the combined cycles were carried out, and it was found that the heat addition process had the maximum irreversibility, followed by the ejector and turbine [11,12]. The ejector performance under the critical mode of operation and an hourly dynamic simulation of the SERS of 7 kW refrigeration capacity was developed using TRNSYS Software coupled with EES (Engineering Equation Solver) [13,14].

An ejector is a device used to suck the vapor from the vessel or framework. The real distinction between the ejector and the vacuum pump or compressor is that it had no moving parts. Hence, it is relatively cheap and easy to operate and almost free to maintain equipment. The primary nozzle location (PNL) determines the type of ejector in terms of constant pressure mixing (CPM) or constant area mixing (CAM). If the PNL is in the downstream location of the suction chamber, then it is a CPM, while if the PNL is in the constant area section, the ejector is CAM. The performance of the CPM ejectors is better than that of the CAM ejector [15]. The main advantages of using ejectors in the systems are no moving parts; minimal to zero maintenance; robust construction; safe to install and upgrade; operates on gas, liquid, and multi-phase; low cost; minimal control and instrumentation; and lower weight.

The ejector was introduced into the VCRS to enhance the system performance and increase its coefficient of performance (COP) [16]. In another work, researchers presented a thermodynamic analysis of an ejector cascade cooling cycle for low and medium temperatures [17,18]. In addition, the implementation of new technologies and cycle modifications was introduced to improve the cycle performance. Mathematical and thermodynamic models of the integrated Rankine power cycle into the ERS were performed to estimate the effect of key parameters on the system performance [19,20,21].

ORC is implemented to produce power from low and medium grade heat sources when the temperature is in the range of 80 to 350 °C. The low-grade heat that could be wasted can be utilized and recovered in these technologies [22]. The circulated organic fluid in the ORC is selected for the best accommodation of the heat source based on their thermodynamic properties to achieve the highest cycle efficiency.

ORC systems are used to harvest low-grade waste heat and, therefore, increase the overall thermal efficiency of the system. The low-grade heat is transformed into useful work in the form of electricity. Simulation of organic Rankine cycle is carried out in EBSILON Professional Software [23].

One of the factors affecting the cycle performance is the working fluid and its thermal and physical properties. The selection processes of the best environmentally friendly working fluid for ORC and ERC cycles were studied by several authors [24,25]. The environment friendly refrigerants that have the best performance characteristics with lower global warming and ozone depletion impacts on the environment were found to be R600, R134a, and R1234yf.

Exergy is the theoretical maximum work that could be obtained in terms of either the shaft or electrical work from an energy system when it is brought into thermodynamic equilibrium with the environment [26]. It measures how different is the actual state of a system in comparison with the environment, which is in thermodynamic equilibrium with total exergy equal to zero, and with no irreversibility. The exergetic performance of the ORC combined with an ERC was thermodynamically studied for the use of a low-grade heat source [27,28]. The results indicated that the losses and destruction in exergy were the highest in the boiler and the lowest in the expansion valve, and the efficiency was better in the case of a low critical temperature working fluid. Other works studied in detail the combined ERS and ORC, which could recycle the waste heat for power and refrigeration, and compared it with the conventional cycle [29,30]. The costs that the owners and/or investors must recover include, but are not limited to preliminary feasibility and engineering studies, development costs, environmental studies, legal fees, taxes, and electrical interconnection costs [31,32]. The efficiency of the ejector components significantly affects the exergy destruction within the components as well as in other components [33,34].

Extensive research works were conducted on the combined cycles’ configuration and economic optimization. The research work concluded that combined cycles for trigeneration of cold, heat, and power with wasted or low-grade heat were efficient [35,36,37]. New combined ERS with ORC were studied, and it was found that the power needed to drive the compressor could be decreased, and the system COP increased [38].

Different types and configurations of air conditioning and refrigeration systems are used in industrial, residential, and commercial applications to maintain comfort conditions. In the case of the application of these systems in places and areas without reliable power supply, the low-grade heat source can be utilized in refrigerating machines, which makes them promising technologies. It has been found in the literature that the ERC has a maximum COP at an optimum generator temperature. In addition, owing to the dynamic behavior of the solar radiation and its variation during the day, the amount of energy utilized by ERC is much less than the total energy harvested. Therefore, in this study, a new configuration ERC configuration combined with an ORC is presented to maximize the use of the collected solar energy and increase the total thermal efficiency of the system. A hybrid autonomous SERS combined with an ORC to produce a refrigeration effect and power is investigated. The ERC is modeled in Engineering Equation Solver Software (EES), and the results were used to model the entire cycle in EBSILON Professional. The ERC refrigeration capacity is 10.75 kW, and the rated power output of the ORC is 0.76 kW. It is intended to maximize the harvesting of the collected solar energy by introducing the ORC into the system so that the ERS utilizes its need from the solar energy collected, and the excess energy will be used in the ORC to produce electrical power. This electricity could be used to power the pumps and the system’s control unit and, therefore, make the system autonomous. A parametric study of the operating conditions’ effect on the cycle performance an exergy study is carried out to evaluate the real thermodynamics inefficiencies within the system. The exergy efficiency and exergy destruction values of the system components are evaluated. The approach of exergoeconomic analysis is used to find the comparative cost importance for each component in the system. It takes into consideration not only the bare module cost, fuel, and operation and maintenance cost (OMC), but also the cost of the exergy destruction within the component. The total revenue requirement (TRR) method is used as a basis for an economic analysis of the system.

## 2. System Overview and Description

**Figure 1:**
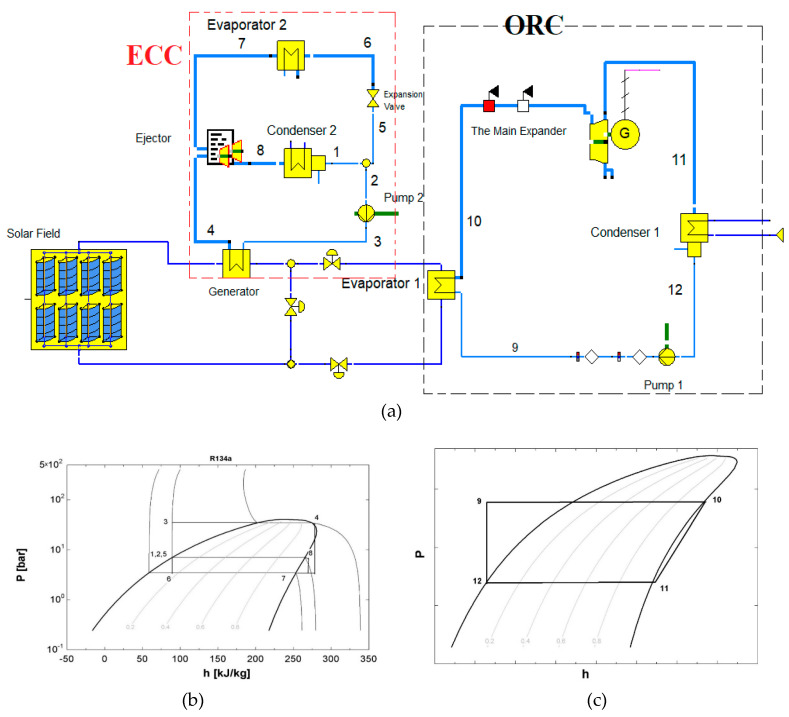
(**a**) The hybrid solar ejector refrigeration cycle (SERC) with organic Rankine cycle (ORC), (**b**) the *lg p-h* diagram of the ERC, and (**c**) the *lg p-h* diagram of the ORC.

The ERS mainly consists of five components: generator, condenser, evaporator, ejector, and the expansion valve. Here, the roles of the turbine and compressor are replaced by the ejector that has no moving parts. The entire system can produce a refrigeration effect using waste heat or solar energy. As shown in Figure 1a, the solar energy is used in the generator to heat the primary flow (Process 3–4). This primary flow continues to flow through the converging-diverging nozzle in the ejector, where it gains speed. The high-speed flow creates a reduction in the pressure and induces a secondary flow to the ejector from the evaporator. The primary and secondary streams combine in the mixing chamber and enter the diffuser. The pressure increases to the condenser pressure and enters the condenser, where the vapor condenses into a liquid. The refrigerant liquid is divided into two streams; one is pumped to the generator, and the other portion is directed to the expansion valve. The refrigerant expands to the evaporator pressure and evaporates, producing the refrigeration effect, and the vapor from the evaporator is sent to the ejector to complete the cycle.

From the solar collector, the accumulated energy in the storage tank is further transferred to the generator. The working fluid in the generator gets heated up and vaporized, and the same process continues in the ERS.

## 3. Methods

### 3.1. Energy Analysis

The ERS is modeled in EES, and a computer program is developed based on the mass, momentum, and energy conservation principles. The energy balances are as follows:

For the evaporator:

(1) $${\dot{Q}}_{e}={\dot{m}}_{e}\left(h_{7}-h_{6}\right)$$

For the generator:

(2) $${\dot{Q}}_{g}={\dot{m}}_{g}\left(h_{4}-h_{3}\right)$$

For the condenser:

(3) $${\dot{Q}}_{c}=\left({\dot{m}}_{p}+{\dot{m}}_{s}\right)\left(h_{8}-h_{1}\right)$$

For the ejector

(4) $$({\dot{m}}_{p}+{\dot{m}}_{s})h_{8}=h_{4}{\dot{m}}_{p}+h_{7}{\dot{m}}_{s}$$

The throttling process is as follows:

(5) $$h_{5}=h_{6}$$

The pump power is as follows:

(6) $${\dot{W}}_{p}={\dot{m}}_{p}\left(h_{3}-h_{2}\right)$$

The performance of the ERS is given by COP as follows:

(7) $$COP={\dot{Q}}_{e}/\left({\dot{Q}}_{g}+{\dot{W}}_{pump}\right)$$

The following assumptions were considered in the modeling process of the cycle:

- Flow-through, the ejector, is 1-D, adiabatic, and steady;
- Isentropic flow through diffuser and nozzle;
- CPM ejectors are used because they generate higher condenser pressures than ejectors of CAM with similar COP and entrainment ratios;
- Generator pressure and temperature are 33 bar and 90 °C, respectively;
- Evaporator pressure and temperature are 4.5 bar and 12.45 °C, respectively;
- The throat diameter of the ejector is 0.000605 m.

**Figure 2:**
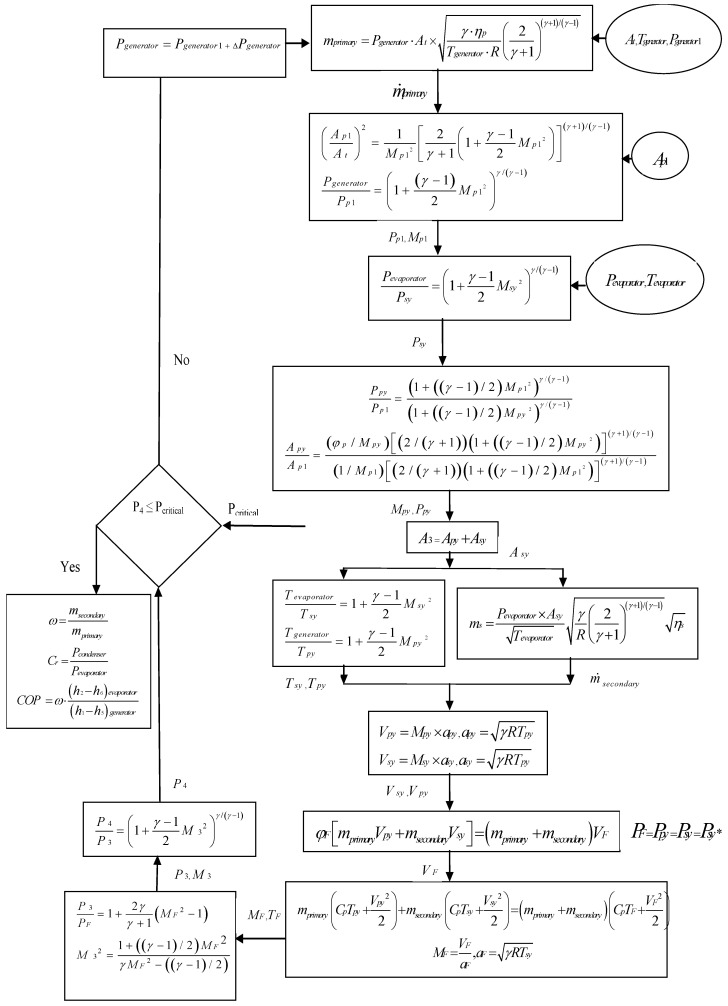
The ejector mathematical model flow chart.

The thermodynamic model of the ejector is presented in the flow chart given in Figure 2. Several values of pressure and temperature of the generator and evaporator were used in the simulation program. The effect of variation of these parameters on the ERC performance was studied.

### 3.2. Exergy Analysis

The energy balance is mainly concerned with the energy quantity, and it does not account for the quality of energy. In thermodynamics, the quality of a given quantity of energy is characterized by its exergy [24].

Exergy analysis is performed as an extension of energy analysis. It is useful because it provides a more accurate and more robust analysis of thermodynamic systems than an energy analysis. Besides, it accounts for the useful energy of an energy stream or the part of energy in an energy stream capable of performing work. Energy analysis merely gives a gross sum of energy regardless of its usefulness. Exergy analysis can be performed on a system level, subsystem level, or component level. Each case provides different information that is useful for system optimization.

In a thermodynamic system, the real inefficiencies are the exergy destruction occurring within the system boundaries and the exergy transferred to the surrounding system (exergy losses). Some of the causes of exergy destruction are a chemical reaction, fluid friction, throttling of flow, mixing of dissimilar flow, and heat transfer through finite temperature difference. The total exergy of the system consists mainly of four components: chemical, physical, potential, and kinetic exergy. The physical exergy is further considered, and all other forms are neglected. The temperature *T*_0_ is 298.15 K and pressure *p*_0_ is 1.013 bar for the reference environment in this analysis.

The exergy calculations were performed on a streaming basis and then on a component basis using the data from EBSILON Professional. The stream basis gives an overview of the exergy carried by each stream. The fuel and product methods are used for the components’ exergy balance and estimation of exergy destruction.

(8) $${\dot{E}}_{F,tot}={\dot{E}}_{P,tot}+{\dot{E}}_{D,tot}+{\dot{E}}_{L,tot}$$

The rate of exergy loss is not considered for individual components, therefore,

(9) $${\dot{E}}_{F,k}={\dot{E}}_{P,k}+{\dot{E}}_{D,k}$$

Exergetic efficiency *ε_k_* is calculated by the rate of exergy of product and exergy of fuel

(10) $$\varepsilon_{k}=\frac{{\dot{E}}_{P,k}}{{\dot{E}}_{F,k}}\;$$

Overall system exergy efficiency *ε_tot_*

(11) $$\varepsilon_{tot}=\frac{{\dot{E}}_{P,tot}}{\;{\dot{E}}_{F,tot}\;}$$

The exergy destruction ratio $y_{D,k}^{*}\;$

(12) $$y_{D,k}^{*}=\frac{{\dot{E}}_{D,k}}{\;{\dot{E}}_{D,tot}\;}$$

There are some components in the system known as dissipative components, such as the throttling valve and condenser. In these components, exergy is destroyed or transferred into the environment without obtaining the positive exergetic effect. No exergetic efficiency can be defined for these components. Only the thermodynamic inefficiency should be calculated for each dissipative component

(13) $${\dot{E}}_{k,dissipative}={\dot{E}}_{in,k}+{\dot{E}}_{out,k}$$

### 3.3. Economic Analysis

The performance characteristics and operating costs are essential for the competitiveness and economic feasibility of a project. The increasing awareness among consumers, regarding health and climate change, leads to social acceptance becoming increasingly crucial for the smooth commissioning of a new system. This acceptance requires the projects to be as efficient and environmentally friendly as possible without becoming exceedingly expensive. Investors have to plan, design meticulously, and analyze the system they want to build and ensure a sound technical efficiency as well as a profitable return on the investment.

For economic analysis, the TRR method for a system is used. The annual system TRR is the annual collected revenue that ensures the compensation of the system operating costs and ascertains the economical operation of the plant. The levelized TRR is the addition of carrying charges (*CC_L_*) and the expenses of fuel and operation and maintenance costs (*FC_L_*) and (*OMC_L_*), respectively.

(14) $$TRR_{L}=CC_{L}+FC_{L}+OMC_{L}$$

The *CC_L_* is the capital investment cost, which includes total capital recovery, preferred stock, return on investment for debt, income taxes, and other taxes and insurances. Expenses are mainly *FC_L_* and *OMC_L_*. The *CC_L_* is the levelized value of the total capital investment cost (*TCI*), which is composed of the fixed capital investment (*FCI*) plus the interest to be paid for the investment.

(15) TCI=FCI+interest

The *FCI* is calculated by adding the cost of the bare module cost (*BMC*) to the service facilities, architectural work, and contingencies. The *FCI* would represent the total system cost if it had a zero-time design and construction period. The indirect system costs are construction costs, contingencies, administrative fees, and engineering.

(16) $$FCI=BMC_{tot}+service\;facilities+architechtural\;work+comntigencies$$

The first step in the estimation of *TCI* was to estimate the purchase equipment cost (*PEC*) using graphs, cost functions, or market research. The second step in the estimation was to adjust the purchase equipment cost for the required size using the power law as follows:

(17) $$C_{PE,new}=C_{PE,known}{\left(\frac{X_{new}}{X_{known}}\right)}^{\alpha }$$

where $C_{PE,new}$ is the approximate equipment costs having the size $X_{new}$, $C_{PE,known}$ is the known equipment costs having the corresponding size $X_{known}$, and α is the size exponent. The dimensionless size exponent α can be found, for example, in [24].

Different methods of cost estimation are based on empirical data. Usually, the values are calculated for a specific year of reference and adapted to the year of analysis using the chemical engineering plant cost index (*CEPCI*) factors [33]. The third step is to update the estimated equipment cost $C_{PE,new}$ to the reference year. The reference year is chosen as 2018, with *CEPCI* equal to 603.1.

(18) $$C_{PE,ref}=C_{PE,old}\;\left(\frac{CEPCI_{ref}}{CEPCI_{old}}\right)$$

where subscript *ref* refers to the year the equipment is supposed to be purchased and subscript *old* refers to the year the cost of equipment was valid.

After calculating the current proportionated equipment purchase cost, the particular nature and characteristics of the equipment have to be considered in the form of factors. For unique materials, high pressures, and distinctive designs, the materials and pressure correction factors (*MPF*) were defined. It can be a function of design variation (subscript *d*), pressure variation (subscript *p*), construction material variation (subscript *m*), and operational limits (subscript *o*). The way of calculating the *MPF* and variations that are considered depend on the equipment. The final step in the cost estimation method was to account for the installation costs using the module factor (*MF*). The *MF* takes into account labor, piping instruments, accessories, and everything necessary for installing the equipment. A typical value for the *MF* is 2.95. The final *BMC* can be calculated with the following equation:

(19) $$BMC=C_{PE,ref,new}\left(MPF+MF-1\right)$$

The total direct costs were calculated as the sum of *BMC*, whereas the indirect costs were calculated as a percentage of the total direct costs. In order to get the *TCI*, the other outlays had to be considered together with the *FCI*. The other outlays consisted of the allowance for funds used during construction, which considered the time value of money and the interest that occurred during the construction period and had to be repaid.

Having calculated the total amount of money that would be invested, the system’s economic life, the interest rate, the average inflation, and the average nominal escalation rate of the fuel had to be considered. These inputs were used to calculate the constant escalation levelization factors (*CELF*) and the capital recovery factor (*CRF*) to perform a detailed cost calculation.

(20) $$CRF=\frac{i_{eff}{\left(1+i_{eff}\right)}^{n}}{{\left(1+i_{eff}\right)}^{n}-1}\;$$

$i_{eff}$ is the effective interest rate, and *n* is the economic lifetime.

$FC_{L}$ is determined as follows:

(21) $$FC_{L}=FC_{0}*CELF=FC_{0}*\frac{k_{FC}\left(1-k_{FC}^{n}\right)}{1-k_{FC}}*CRF$$

and

(22) $$OMC_{L}=OMC_{0}*CELF=OMC_{0}*\frac{k_{OMC}\left(1-k_{OMC}^{n}\right)}{1-k_{OMC}}*CRF\;$$

with $k_{FC}=\frac{1+r_{FC}}{1+i_{eff}}$ and $k_{OMC}=\frac{1+r_{OMC}}{1+i_{eff}}$, where $r_{FC}$ is the average inflation rate of the fuel cost and $r_{OMC}$ is the operating and maintenance cost. $FC_{0}$ is the first-year fuel cost, and CELF is the constant escalation levelization factor.

### 3.4. Exergoeconomic Analysis

Exergoeconomic analysis is a combination of exergy and costs analyses to present to the designer or the operator of an energy conversion system with the information not available through conventional energy, exergy, or cost analysis. This approach of exergoeconomic is used to find the comparative cost importance for each component in the system. The information obtained through exergy and economic analyses are combined to determine the cost of each stream in the system. The main aim is to create a cost balance of each component in the system.

For each component of the system,

(23a) $${\dot{C}}_{P,k}={\dot{C}}_{F,k}+{\dot{Z}}_{k}$$

(23b) $$c_{P}{\dot{E}}_{P,k}=c_{F}{\dot{E}}_{F,k}+{\dot{Z}}_{k}$$

where the average component cost of fuel $c_{F}=\frac{{\dot{C}}_{F,k}}{{\dot{E}}_{F,k}}$ and product $c_{P}=\frac{{\dot{C}}_{P,k}}{{\dot{E}}_{P,k}}$, and the exergy destruction cost rates within the component,

(24) $${\dot{C}}_{D,k}=c_{F}{\dot{E}}_{D,k}$$

For the overall system,

(25a) $${\dot{C}}_{P,tot}={\dot{C}}_{F,tot}+{\dot{Z}}_{tot}-{\dot{C}}_{L,tot}$$

(25b) $$c_{P,tot}{\dot{E}}_{P,tot}=c_{F,tot}{\dot{E}}_{F,tot}+{\dot{Z}}_{tot}-{\dot{C}}_{L,tot}$$

where the average total cost of fuel $c_{F,tot}=\frac{{\dot{C}}_{F,tot}}{{\dot{E}}_{F,tot}}$ and product $c_{P,tot}=\frac{{\dot{C}}_{P,tot}}{{\dot{E}}_{P,tot}}$, and the exergy destruction cost rates within the component,

(26) $${\dot{C}}_{D,tot}=c_{F,tot}\sum {\dot{E}}_{D,k}$$

Exergoeconomic factor *f_k_* indicates the relative contribution of the exergy destruction cost rate and those associated with the *CC_L_* and *OMC*. This factor is used during the optimization to make decisions of whether to invest in a more efficient component to reduce the exergy destruction or to sacrifice efficiency to decrease the cost rate associated with the carrying charges.

(27) $$f_{k}=\frac{{\dot{Z}}_{k}\;}{\;{\dot{Z}}_{k}+{\dot{C}}_{D,k}}$$

## 4. Results and Discussion

The entire system cycle represents the combination of the SERC with the ORC, where solar energy is used to drive both cycles for cogeneration of cooling capacity and electrical power. The simulation of the ERC and ORC was carried out individually, and later, the solar subsystem, which includes solar collectors and hot storage tank, was added to the primary cycle, and the whole cycle was designed in EBSILON Professional.

The assumptions used for the solar subsystem are that the solar collector is an evacuated tube collector (50 m^2^), outlet temperature of collector = 111.1 °C, maximum storage capacity = 2000 kg, storage volume = 2 m^3^, storage pressure = 10 bar, average storage temperature = 110 °C, and direct normal irradiation (DNI) = 1100 W/m^2^.

**Figure 3:**
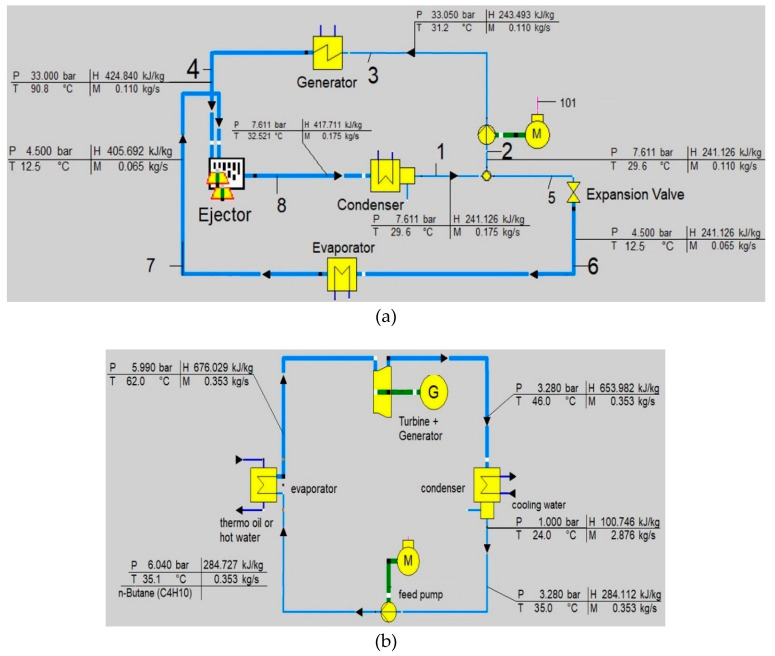
Schematic and the thermodynamic parameters for the ejector refrigeration cycle (**a**) and organic Rankine cycle (**b**).

The obtained values of the thermodynamic parameters for the SERC and organic Rankine cycle are shown in Figure 3. After having the results of all the parameters of the ejector refrigeration cycle in the EES, it is further modeled in EBSILON Professional Software. The results show an excellent agreement with those data obtained from the EES. The refrigeration capacity of the ejector refrigeration cycle is 10.75 kW.

For the simulation of the ORC in EBSILON Professional, the specification of the parameters was taken from the model reported in [10]. The simulation results of the studied model are compared with those of published data [10] given in Table 1.

**Figure 4:**
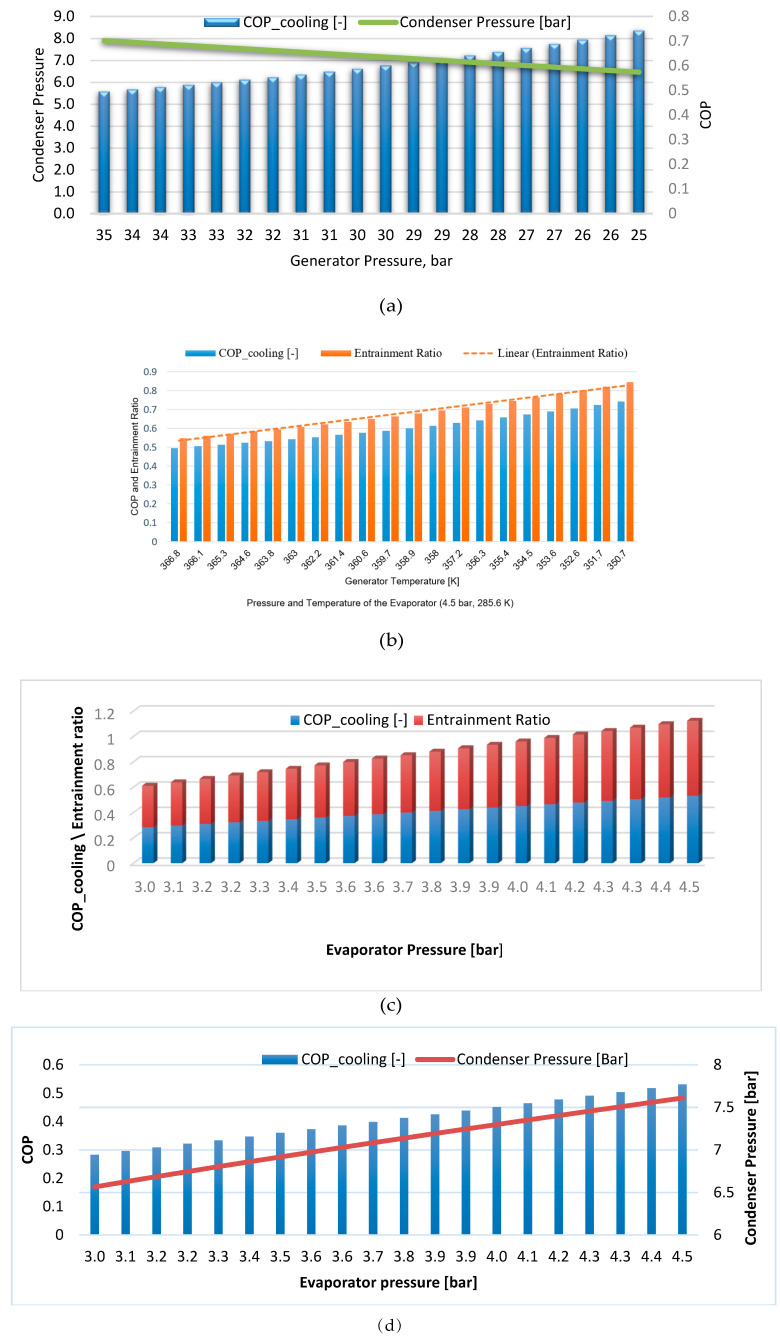
Effect of generator pressure on (**a**) the coefficient of performance (COP) and condenser pressure; (**b**) the COP and entrainment ratio; the effect of evaporator pressure on (**c**) the COP and entrainment ratio; and (**d**) the COP and condenser pressure, Pg = 33 bar, Tg = 363.9 K, respectively.

Figure 4 shows the results obtained for the SERC to evaluate the system performance.

The simulations for different generator pressures (Figure 4a,b) show the following. As the generator pressures decrease, with the evaporator temperature and pressure, and are constant at 285.6 K and 4.5 bar, respectively, the condenser pressure decreases, and the *COP* increases. It was found that a decrease of 28% in the generator pressures results in a 48% increase in the cycle *COP* and a decrease of 18% in the condenser pressure. Figure 4b shows the change in the *COP* and the entrainment ratio as the generator temperature varies, while keeping the evaporator temperature and pressure constant at 285.6 K and 4.5 bar, respectively. As the generator temperature is decreased from 366.8 K to 350.7 K, the *COP* of the cycle is increased from 0.5 to 0.75, and the entrainment ratio also increases from 0.55 to 0.85. Thus, by decreasing the generator tepmerature, the *COP* of the system is increased, and the entrainment ratio is also increased.

As shown in Figure 4c, the effect of evaporator pressure on the *COP* and entrainment ratio is given at a constant pressure and temperature within the generator, 33 bar and 363.9 K, respectively. These figures depict the effect on the *COP* and entrainment ratio by changing evaporation pressure from 3 bar to 4.5 bar. The values of *COP* and the entrainment ratio are both increased by increasing the evaporator pressure.

Figure 4d shows the effect of evaporator pressure on the *COP* and the condenser pressure at constant generator pressure and temperature. The *COP* of the system is increased from 0.28 to 0.53 for the evaporator pressure change, and the condenser pressure increases as well.

**Figure 5:**
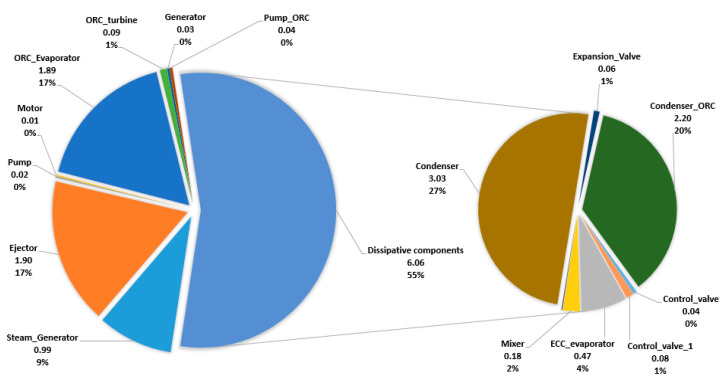
Exergy destruction [kW] and exergy destruction ratios [%] within the group of productive and dissipative components. ORC, organic Rankine cycle, Ejector Cooling Cycle ECC.

**Figure 6:**
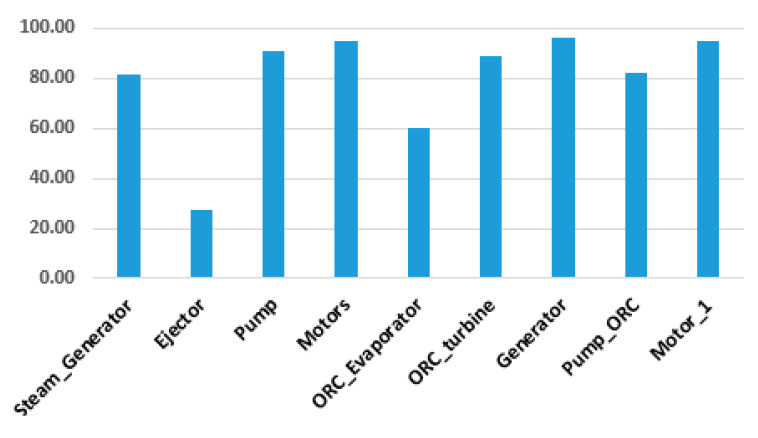
Exergetic efficiency [%] for the product components.

The exergy analysis was conducted in the EBSILON Professional Software, while the only physical exergy of material streams should be considered (Figure 3a, Table 2). The values of the exergy destruction and exergy destruction ratios are shown in Figure 5, and the exergy efficiency for productive components is shown in Figure 6.

The results obtained from the exergetic analysis indicate that the condensers (of the ERC and the ORC, both are dissipative components) have higher exergy destruction values, followed by the ejector in the ERC, the ORC evaporator, and the steam generator, as shown in Figure 5. The solar field has, by far, the highest exergy destruction (not shown in Figure 5). The operation conditions of the condenser have the most substantial influence on the destruction of exergy and the overall system. The highest exergetic efficiency is in the case of generator, pumps, and motors, while the lowest exergetic efficiency is in the case of the ejector and the solar field. The exergetic efficiency of productive components, except the ejector, is quite high. The total exergetic efficiency of the overall system is around 20%.

**Figure 7:**
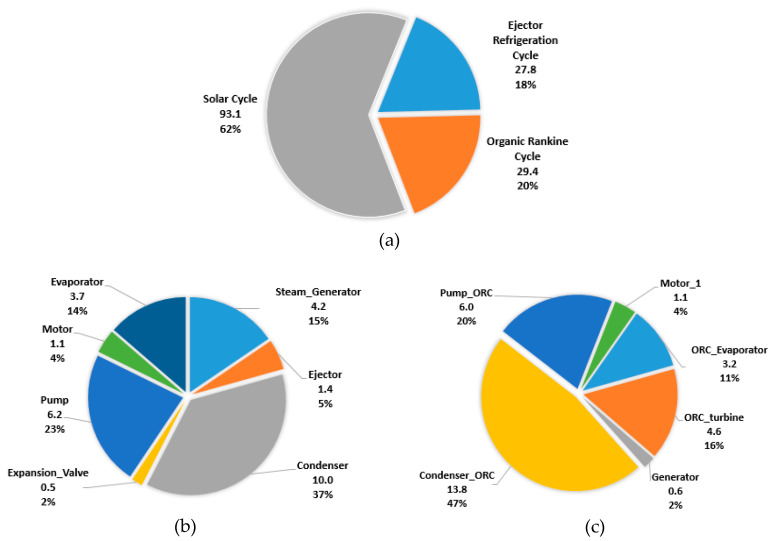
Bare module cost (BMC) (×10^3^ USD) share of the system (**a**) and individual components of ERC (**b**) and ORC (**c**).

Table 3 shows the assumptions used to conduct the economic analysis. A detailed *BMC* of each system component is calculated and is shown in Figure 7a,b for the ERC and ORC, respectively, where materials and pressure factors were considered. The majority of the components were chosen to be made of carbon steel or stainless steel. The total *BMC* of the system is found to be around 150.1 × 10^3^ USD. The levelized carrying charges (CCL) are 31.7 × 10^3^ USD/year, and the levelized operation and maintenance costs (OMCL) are 9.9 × 10^3^ USD/year. As expected, the highest *BMC* is of the solar field or collector and the storage tanks. The resulting levelized cost of electricity (*LCOE*) for the system being evaluated is 1.8 USD/kWh.

In order to proceed with the exergoeconomic analysis, the values of cost rate associated with the capital investment within the component (${\dot{Z}}_{k}$) should be calculated as

(28) $${\dot{Z}}_{k}=\frac{\left(\left(CC_{L}+OMC_{L}\right)*BMC_{k}\;\right)}{\left(Annual\;time\;of\;operation*C_{BMC_{TOT}}\right)}\;$$

Cost balances (and, if required, the auxiliary equations) were formulated for each system component. Cost rates associated with exergy destruction (*Ċ_D,k_*) and exergoeconomic factors (*f_k_*) were calculated.

**Figure 8:**
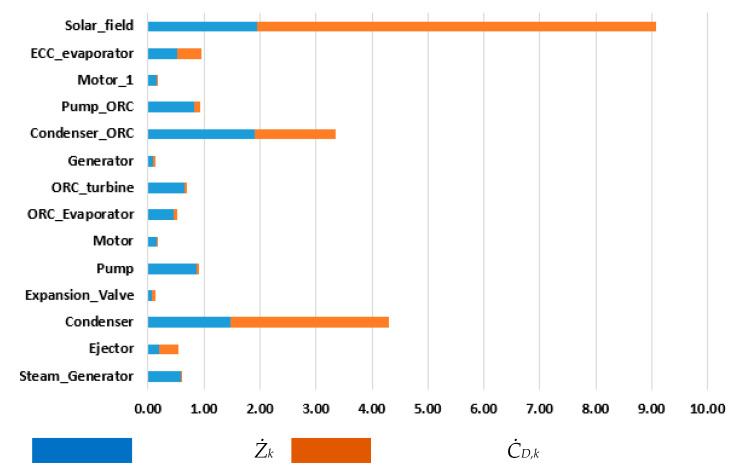
The sum of (*Ż_k_* + *Ċ_D,k_*) for the components of the overall system (USD/h).

**Figure 9:**
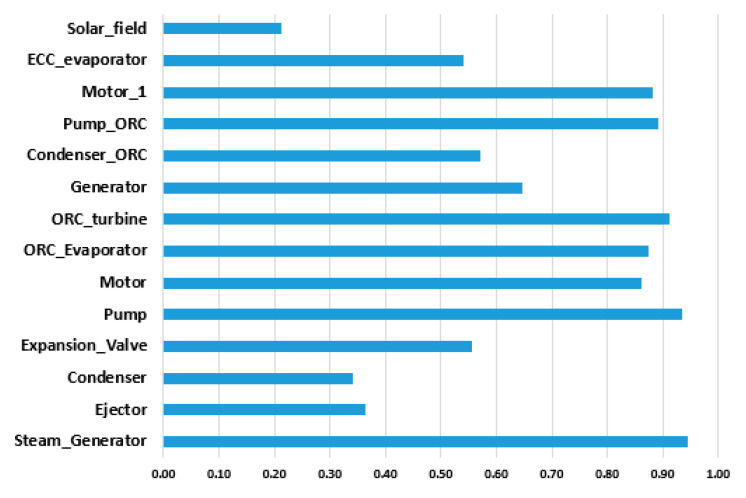
Exergoeconomic factor (*f_k_*) of system components.

Figure 8 shows the sum of (*Ż_k_* + *Ċ_D,k_*) for the components of the overall system, and Figure 9 shows the exergoeconomic factor of these components. High exergoeconomic factor values of a component suggest a decrease in the investment costs of that component regardless of its exergetic efficiency.

The highest sum (*Ż_k_* + *Ċ_D,k_*) is for the solar field, followed by the condenser of the ERC and the condenser of the ORC. The exergoeconomic factor of the solar field is 21%, suggesting that the capital cost is a relatively much lower rate than the cost of exergy destruction. So, 79% of the total cost associated with the solar field is owing to its high thermodynamic inefficiency (high exergy destruction). The second cost-ineffective component in the system is found to be the condenser, and its exergoeconomic factor is observed to be 34%, which means that only 34% of the cost is owing to component capital cost and 66% of the cost is because of high exergy destruction. The third cost-ineffective component in the system is found to be the condenser of the ORC, and its exergoeconomic factor is obtained to be approximately 58%, which means that only 58% of the cost is owing to component capital cost and 42% of the cost is owing to high exergy destruction.

## 5. Conclusions

In this work, the ERS is combined with the ORC, and the main supply of heat for both cycles is solar energy. The simulation of the cycle was carried out in EBSILON Professional Software. The ERS is modeled in EES, and the results are taken to model the ERS in EBSILON Professional.

Exergy analysis is carried out to find out the thermodynamics inefficiencies in the system. The solar field has the highest exergy destruction, which is attributed to heat transfer across a finite temperature difference, mixing, and fluid friction. It was found that the condenser of the ERS had the highest exergy destruction, followed by condenser of the organic Rankine cycle, ejector in the ejector ERC, ORS evaporator, and steam generator. The total exergetic efficiency of the system obtained is 20%.

For economic analysis, the TRR method for a system was applied. The total BMC of all system components amounted to 150.1 × 103 USD.

The exergoeconomic analysis was used to find the comparative cost importance for each component in the system. The solar field has the highest cost associated with the component (capital cost and the cost of exergy destruction) and the lowest exergoeconomic factor, suggesting that modern high efficient solar technology should be used for such combined ERS/ORC systems.

## Figures and Tables

**Table 1:** Comparison of organic Rankine cycle (ORC) models.

Variable	Nomenclature	Unit	Model in [10]	**Study Model**
**Working fluid**			n-Butane	n-Butane
**Heat in**	${\dot{Q}}_{in}$	kW	138.17	138.13
**Expander inlet**	$\dot{m}$	kg (s)^−1^	0.353	0.353
	*p*	bar	5.99	5.99
	*T*	°C	62.0	62.0
**Expander outlet**	$\dot{m}$	kg (s)^−1^	0.353	0.353
	*p*	bar	3.28	3.28
	*T*	°C	44.19	45.00
**Condenser inlet**	$\dot{m}$	kg (s)^−1^	0.353	0.353
	*p*	bar	3.28	3.28
	*T*	°C	44.1	44.0
**Condenser outlet**	$\dot{m}$	kg (s)^−1^	0.353	0.353
	*p*	bar	3.28	3.28
	*T*	°C	35.0	35.0
**Expander power**	${\dot{W}}_{ex}$	kW	8.37	8.49
**Pump power**	${\dot{W}}_{p}$	kW	0.17	0.17
**Net power**	${\dot{W}}_{\mathrm{net}}$	kW	8.20	8.31
**Energetic efficiency**	*η*	%	5.93	6.14

**Table 2:** Exergy rate for material streams.

State	Exergy Rate [kW]	State	Exergy Rate [kW]	State	Exergy Rate [kW]
**1**	4.36	**10**	4.35	**19**	7.46
**2**	2.74	**11**	2.15	**20**	7.42
**3**	2.97	**12**	2.33	**21**	9.64
**4**	7.25	**13**	2.65	**22**	9.56
**5**	1.62	**14**	12.04	**23**	3.41
**6**	1.56	**15**	18.07	**24**	5.79
**7**	2.03	**16**	75.94	**25**	1.78
**8**	7.39	**17**	22.37	**26**	3.66
**9**	5.20	**18**	14.91		

**Table 3:** Parameters and assumptions for economic analysis. CRF, capital recovery factor; CELF, constant escalation levelization factor.

Parameters/Assumptions	Value
**Plant economic life**	20 years
**Effective interest rate**	10%
**CRF**	0.117
**Average general inflation rate**	4.5%
**Average nominal escalation rate of fuel costs**	1.7%
**CELF general**	1.2143
**CELF fuel**	1.3171
**Annual full load operational time**	2000 h

## Nomenclature

$\dot{C}$	cost rate [USD (h)^−1^]
*COP*	coefficient of performance [-]
$\dot{E}$	exergy rate [W]
*f*	exergoeconomic factor [-]
*h*	specific enthalpy [kJ (kg)^−1^]
*d*	throat diameter [-]
*i*	interest rate [%]
$\dot{m}$	mass flow rate [kg (s) ^−1^]
*n*	economic life [year]
*p*	pressure [bar]
$\dot{Q}$	heat rate [W]
*r*	inflation rate [%]
*T*	temperature [K, ^o^C]
$\dot{W}$	power [W]
*y^*^_D_*	exergy destruction ratio [%]
$\dot{Z}$	cost rate [USD (h)^−1^]
*Greek symbols*	
α	exponent for size component
*ε*	exergy efficiency [%]
τ	annual operating hours [h (year)^−1^]
ω	entrainment ratio [-]
*η*	efficiency [%]
*Abbreviations*	
AC	air conditioning
BMC	bare module cost
CAM	constant area mixing
CC	carrying charges
CELF	constant escalation levelization factor
CPM	constant pressure mixing
CRF	capital recovery factor
DNI	direct normal irradiation
ERC	ejector refrigeration cycle
ERS	ejector refrigeration system
FC	fuel cost
FCI	fixed capital investment
LCOE	levelized cost of electricity
MF	module factor
MPF	material and pressure correction factor
OMCL	levelized operation and maintenance cost
ORC	organic Rankine cycle
PEC	purchased equipment cost
PNL	primary nozzle location
SERC	solar ejector refrigeration cycle
TCI	total capital investment
TRR	total revenue requirement
VCRS	vapor compression refrigeration system
*Subs- and superscripts*	
*0*	reference state (for exergy analysis)
*D*	exergy destruction
*F*	exergy of fuel
*k*	*k*th component
*L*	exergy losses, levelized
*P*	product
*tot*	overall system
